# The Use and Abuse of Soap

**Published:** 1907-12-07

**Authors:** 


### Dermatology.

### THE USE AND ABUSE OF SOAP.

Apart from the manifold surface-impurities derived from the atmosphere and from fomites, the secretions of the skin itself need the frequent assistance of soap and water for their adequate removal. The cleansing power of this commodity depends upon the extent to which it is able to emulsify all fatty matter by the splitting off of just the right quantity of alkali when it is dissolved in water. While the emulsifying power of soap must be sufficient for this purpose, it is most important that no injury be inflicted upon the living epidermic cells. The most extraordinary ideas are rife with regard to the kind of toilet soap that is used for the daily ablutions, the most unsuitable varieties being constantly employed, until, at last, the human epidermis, which is generally most long-suffering, can stand it no longer, and so irritation of the skin is gradually set up.

### Some Harmful Effects.

Cutaneous troubles resulting from soap depend upon three principal factors: (1) the amount of free alkali present; (2) the presence of some other "foreign" ingredient; and (3) individual susceptibility. A soap containing more than a trace of free alkali is very apt to cause irritation of the skin, as manfested by varying degrees of erythema, which may lead on to a condition of actual eczema. The delicate skin of the face suffers most in this way in proportion to the length of time in which it is in contact with the soap. The superficial layers of the epidermis become eroded, so that the cells beneath fall an easier prey to the effects of changes in temperature, the action of irritants, or the invasion of micro-organisms. Inferior forms of fat, animal or vegetable, in addition to imparting an unpleasant sense of greasiness to the soap, may favour the growth of germs in the skin, and, by hindering the proper emulsification of fatty matter and dirt, will lead towards the retention of the cutaneous secretions with consequent disease. The very doubtful quality of "transparency" may be due to the presence of sugar, and not to glycerine, as thought. This would be distinctly harmful in affections of an eczematous type.

It may be laid down as an axiom that all soap is more or less contra-indicated in acute eczema, and even in the chronic form of the disease the selection of the soap to be used needs to be made with great care. It is an unfortunate and much-to-be-regretted fact that soaps which profess to "cure all skin diseases" are still widely advertised, command a ready sale, and, as often as not, only increase the cutaneous irritation of the suffering purchaser. Soaps which are welcomed in seasons of "spring-cleaning" are quite commonly used by mothers and nurses for scouring the delicate skins of young and tender infants because they are "so nice and cleansing," whereas they are only fit for scrubbing floors. Strong carbolic and tar soaps have an immense popular reputation for curing skin diseases, from eczema to ringworm. The use of soft-soap is quite legitimate in cases of bad seborrhœa capitis, and in eruptions where there is much scaling and crusting, as in psoriasis or impetigo.

It has been found, by actual experiment, that the best all-round soap is obtained by the incorporation of one part of alkali to about eight parts of fatty matter, but these proportions vary in different makes of soap, according to the purpose for which they are intended. The idea of employing soap as a therapeutic agent originated with Hebra, and was amplified by Auspitz; but it was reserved for Unna and Eichoff to suggest and formulate what are now known as neutral soaps, and to lay down the principle of superfatting. A strongly alkaline soap in the form of the familiar Spiritus saponis kalinus (Hebra) is useful for its macerating effect upon the horny layer, but its action requires to be carefully watched, lest the destructive effect becomes too pronounced. Superfatted soaps containing cold cream, with or without oatmeal, may be employed with caution in all but the most acute forms of eczema, for they tend to prevent cracking of the horny layer. The great test of the action of any toilet soap, as Jamieson has pointed out, is to observe its effects upon the skin of a young infant.

### Medicated Soaps.

The so-called "medicated" soaps may be divided into two groups: (*a*) those that are used merely as prophylactics, such as the various disinfectant soaps containing carbolic, sanitas, izal, etc.; (*b*) the really therapeutic soaps, which generally contain an active ingredient to the extent of ten per cent. There still exists some prejudice with regard to the principle of soap-therapy, but it will at once be seen that this method of applying drugs to the skin is cleanly, convenient, and decidedly economical as compared with the use of ointments. A certain amount of penetration of the medicament is bound to occur, and this may be intensified by covering the area with gutta-percha tissue. The use of a superfatted ichthyol soap (10 per cent.) is nearly always attended with considerable improvement in acne and rosacea, while balsam of Peru and sulphur can be similarly incorporated in a soap for the treatment of scabies. Anthrasol, a colourless form of tar, may be employed with advantage in cases of psoriasis or chronic eczema. Whatever variety be chosen, the affected areas may be simply washed with the soap, the lather may be allowed to dry in, more or less vigorous rubbing may be undertaken, or an impermeable covering may be placed over them afterwards, according to the effect it is desired to produce. The application of remedies in this manner is certainly less unpleasant to the patient than when sticky ointments are used, and, if thoroughly carried out, it is quite as efficacious.

